# Inactive matrix Gla protein is a novel circulating biomarker predicting retinal arteriolar narrowing in humans

**DOI:** 10.1038/s41598-018-33257-6

**Published:** 2018-10-10

**Authors:** Fang-Fei Wei, Qi-Fang Huang, Zhen-Yu Zhang, Karel Van Keer, Lutgarde Thijs, Sander Trenson, Wen-Yi Yang, Nicholas Cauwenberghs, Blerim Mujaj, Tatiana Kuznetsova, Karel Allegaert, Harry A. J. Struijker-Boudier, Peter Verhamme, Cees Vermeer, Jan A. Staessen

**Affiliations:** 1 Studies Coordinating Centre, Research Unit Hypertension and Cardiovascular Epidemiology, Department of Cardiovascular Sciences, University of Leuven, Leuven, Belgium; 2 Department of Ophthalmology, University Hospitals Leuven, Leuven, Belgium; 3 Division of Cardiology, University Hospitals Leuven, Leuven, Belgium; 4 Research Unit Organ Systems, Department of Development and Regeneration, University of Leuven, Leuven, Belgium; 5 Department of Pediatric Surgery and Intensive Care and Neonatology, Erasmus Medical Centre, Sophia Children’s Hospital, Rotterdam, The Netherlands; 6 Department of Pharmacology, Maastricht University, Maastricht, The Netherlands; 7 Centre for Molecular and Vascular Biology, Department of Cardiovascular Sciences, University of Leuven, Leuven, Belgium; 8 R&D Group VitaK, Maastricht University, Maastricht, The Netherlands; 9 Cardiovascular Research Institute Maastricht (CARIM), Maastricht University, Maastricht, The Netherlands

## Abstract

Active matrix Gla protein (MGP), a potent inhibitor of calcification in large arteries, protects against macrovascular complications. Recent studies suggested that active MGP helps maintaining the integrity of the renal and myocardial microcirculation, but its role in preserving the retinal microcirculation remains unknown. In 935 randomly recruited Flemish participants (mean age, 40.9 years; 50.3% women), we measured plasma desphospho-uncarboxylated MGP (dp–ucMGP), a marker of poor vitamin K status using an ELISA-based assay at baseline (1996–2010) and retinal microvascular diameters using IVAN software (Vasculomatic ala Nicola, version 1.1) including the central retinal arteriolar (CRAE) and venular (CRVE) equivalent and the arteriole-to-venule ratio (AVR) at follow-up (2008–2015). CRAE (*P* = 0.005) and AVR (*P* = 0.080) at follow-up decreased across tertiles of the dp–ucMGP distribution. In unadjusted models, for a doubling of dp–ucMGP at baseline, CRAE and AVR at follow-up respectively decreased by 1.40 µm (95% confidence interval [CI], 0.32 to 2.48; *P* = 0.011) and 0.006 (CI, 0.001 to 0.011; *P* = 0.016). In multivariable-adjusted models accounting for sex, baseline characteristics and follow-up duration, these estimates were −1.03 µm (CI, −1.96 to −0.11; *P* = 0.028) and −0.007 (CI, −0.011 to −0.002; *P* = 0.007). Additional adjustment for changes from baseline to follow-up in major baseline characteristics yielded as estimates −0.91 µm (CI, −1.82 to −0.01; *P* = 0.048) and −0.006 (95% CI, −0.011 to −0.001; *P* = 0.014), respectively. Circulating inactive dp–ucMGP is a long-term predictor of smaller retinal arteriolar diameter in the general population. Our observations highlight the possibility that vitamin K supplementation might promote retinal health.

## Introduction

Non-mydriatic retinal photography allows non-invasive visualisation of the retinal microvasculature in population surveys^1–4^. Numerous studies demonstrated that the diameters of the retinal microvessels carry important prognostic information^5–7^, smaller arteriolar diameter^6,7^, wider venular calibre^7^, and lower arteriolar-to-venular diameter ratio^5^ predicting cardiovascular mortality^6^, coronary heart disease^5^ and lacunar stroke^7^.

Matrix Gla protein (MGP) is a 11-kD protein synthesised by vascular smooth muscle and endothelial cells^8^. Activation of MGP requires two posttranslational modifications: vitamin-K dependent γ-glutamate carboxylation and serine phosphorylation^8^. Inactive desphospho-uncarboxylated MGP (dp-ucMGP) is a marker of poor vitamin K status^9,10^. Once activated, MGP is a potent locally acting inhibitor of calcification in large arteries^8^ and protects against macrovascular complications^11^ and arterial stiffening^10,12^. MGP is expressed in renal^13,14^ and myocardial microvessels^15^, where the activated protein contributes to maintaining organ function. Similarly, MGP is abundantly expressed in the eye^16–19^, where it takes part in preserving the structural integrity of the trabecular meshwork (TM)16,17, the sclera^18^ and the retinal ganglion cells^19^. A naturally fluorescent *MGP* transgenic mouse model also demonstrated MGP expression in the retinal vasculature^20^. In view of these observations^13–20^, we hypothesised that retinal microvascular traits, as exemplified by retinal arteriolar and venular diameters, might be associated with inactive dp-ucMGP. We assessed in the Flemish Study on Environment, Genes, and Health Outcomes (FLEMENGHO)^11,14^ whether circulating inactive dp-ucMGP predicted retinal microvascular diameters 11 years after the measurement of the biomarker.

## Results

### Characteristics of participants

All 935 participats were White Europeans, of whom 470 (50.3%) were female. The study population consisted of 138 singletons and 797 related subjects, belonging to 164 one-generation families and to 101 multi-generation pedigrees. In all participants, mean baseline values (SD) were 40.9 (14.6) years for age, 122.6 (14.9) mm Hg and 76.4 (11.0) mm Hg for systolic and diastolic blood pressure, and 25.1 (4.3) kg/m^2^ for body mass index. At baseline, 108 participants were on antihypertensive drug treatment, of whom 41 (4.4%) were taking diuretics, 87 (9.3%) inhibitors of the renin-angiotensin system, and 15 (1.6%) vasodilators. At baseline, the geometric mean of dp-ucMGP was 3.45 μg/L and the interquartile range encompassed 2.50 μg/L and 5.16 μg/L. The median interval between baseline and follow-up was 11.0 years (interquartile range, 9.0–13.3 years). In all participants, central retinal arteriolar (CRAE) and venular (CRVE) equivalent and arteriole-to-venule ratio (AVR) at follow-up averaged 150.6 (14.0) µm, 218.3 (19.0) µm and 0.69 (0.06), respectively. Furthermore, the prevalence of glaucoma was about 1% in participants over 40 years old. There were no statistically significant differences in plasma levels of dp-ucMGP and retinal vascular diameters between the five patients with glaucoma and the 518 participants aged 40 years or above without glaucoma (*P* ≥ 0.41).

### Unadjusted analyses

**Figure 1:**
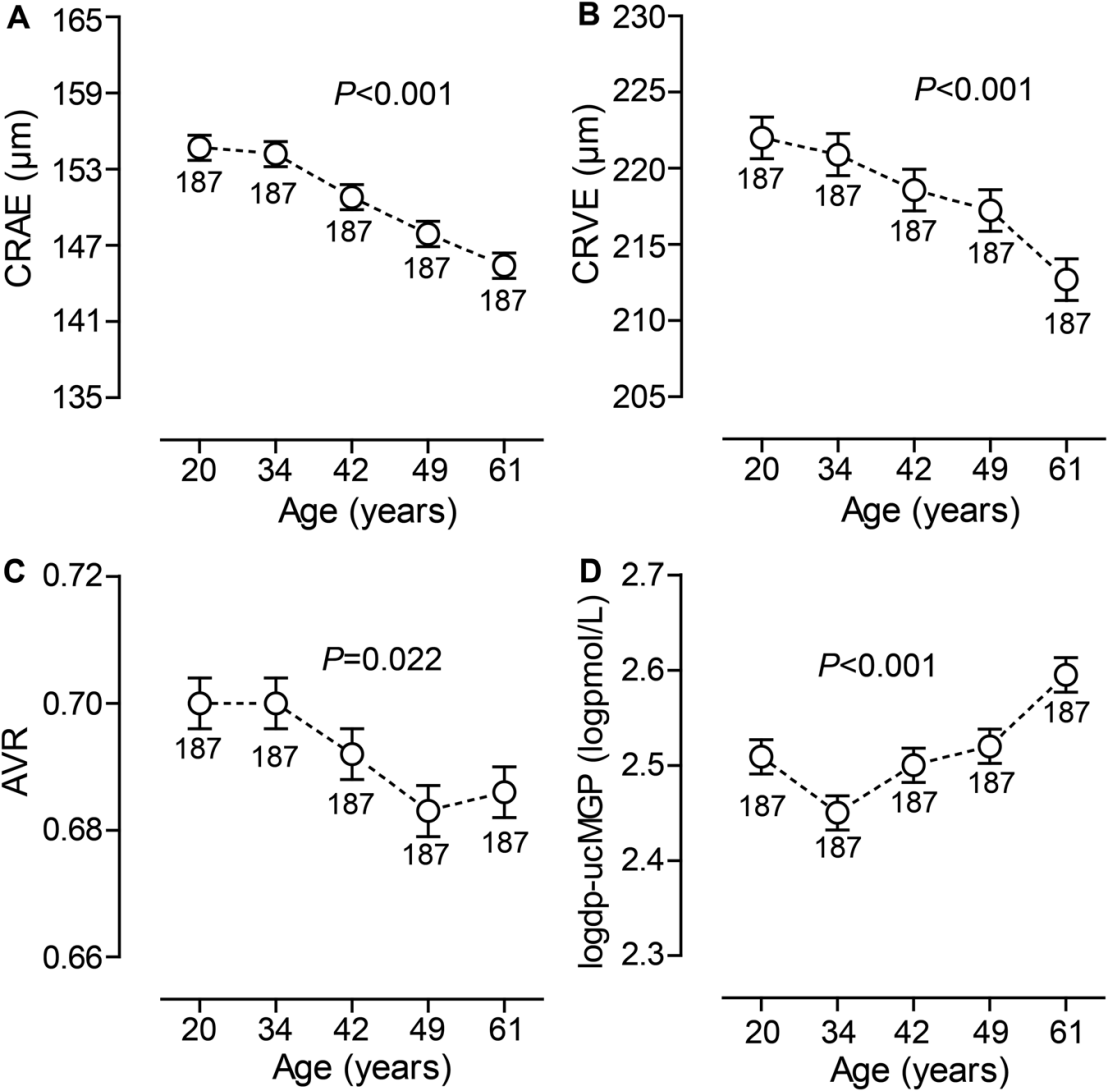
Central retinal arteriolar (CRAE, **A**) and venular (CRVE, **B**) equivalent and arteriole-to-venule ratio (AVR, **C**) at follow-up and baseline inactive desphospho-uncarboxylated matrix Gla protein (dp-ucMGP, **D**) by quintiles of the distribution of baseline age. Vertical bars indicate standard errors. *P*-values are for linear trend across the quintiles of baseline age.

Across tertiles of the baseline dp-ucMGP (Table 1), age, body mass index, blood pressure, total cholesterol and the frequency of hypertension increased (*P* ≤ 0.006), but high-density lipoprotein (HDL) cholesterol and the prevalence of smoking decreased (*P* ≤ 0.037). Table 2 lists the retinal microvascular traits of participants by tertiles of the distribution of dp-ucMGP. CRAE (*P* = 0.005) and AVR (*P* = 0.080) decreased with higher category of dp-ucMGP. Figure 1 demonstrates that in unadjusted analyses dp-ucMGP increased (*P* < 0.001) across fifths of the distributions of baseline age, whereas CRAE, CRVE and AVR decreased (*P* ≤ 0.022).

**Table 1:** Baseline characteristics of participants by tertiles of the dp-ucMGP distribution.

Characteristics	Category of dp-ucMGP			*P* Value
Limits (μg/L)	<2.88	2.88–4.58	≥4.58	
Number of participants (%)	311 (33.3)	311 (33.3)	313 (33.4)	
All patients in category				
Women	166 (53.4)	148 (47.6)	156 (49.8)	0.35
Smokers	88 (28.3)	86 (27.6)	36 (11.5)^‡^	<0.001
Drinking alcohol	133 (42.8)	125 (40.2)	138 (44.1)	0.60
Hypertension	61 (19.6)	61 (19.6)	99 (31.6)^‡^	<0.001
Antihypertensive treatment	27 (8.7)	28 (9.0)	53 (16.9)^†^	0.001
Diabetes mellitus	12 (3.9)	6 (1.9)	9 (2.9)	0.36
Mean (SD) of characteristic				
Age (years)	39.2 (12.3)	39.4 (14.7)	44.1 (16.0)^‡^	<0.001
Body mass index (kg/m^2^)	24.3 (3.8)	24.7 (4.0)	26.3 (4.8)^‡^	<0.001
Systolic pressure (mm Hg)	120.3 (13.0)	121.6 (14.8)	125.8 (16.1)^‡^	<0.001
Diastolic pressure (mm Hg)	75.7 (10.0)	75.3 (11.3)	78.0 (11.6)^†^	0.006
Serum total cholesterol (mmol/L)	5.11 (0.97)	5.04 (0.93)	5.29 (1.06)^‡^	0.004
Serum HDL cholesterol (mmol/L)	1.45 (0.40)	1.46 (0.41)	1.38 (0.34)^*^	0.037
Plasma glucose (mmol/L)	5.03 (1.08)	5.03 (1.09)	5.18 (1.14)	0.14
Geometric mean (IQR) of characteristic				
dp-ucMGP (μg/L)	1.84 (1.56–2.50)	3.64 (3.30–4.04)^‡^	6.10 (5.15–6.79)^‡^	<0.001

Abbreviations: dp-ucMGP, desphospho-uncarboxylated matrix Gla protein. Baseline refers to the date of blood collection for dp-ucMGP measurement. To convert dp-ucMGP from μg/L into pmol/L, multiply by 94.299. Hypertension was a blood pressure of ≥140 mm Hg systolic or ≥90 mm Hg diastolic or use of antihypertensive drugs. Diabetes mellitus was a fasting plasma glucose of ≥126 mg/dL (7.0 mmol/L) or use of antidiabetic agents. *P-*values denote the significance of the difference in prevalence (chi-squared test) or means (ANOVA) across tertiles of the distribution of dp-ucMGP. Significance of the difference with the adjacent lower third: ^*^P ≤ 0.05; ^†^*P* ≤ 0.01; ^‡^*P* ≤ 0.001.

**Table 2:** Retinal microvascular traits by tertiles of the dp-ucMGP distribution at baseline.

Characteristics	Category of dp-ucMGP			*P* Value
Limits (μg/L)	<2.88	2.88–4.58	≥4.58	
Central retinal arteriolar calibre (µm)	152.1 (14.3)	151.0 (13.9)	148.8 (13.5)	0.005
Central retinal venular calibre (µm)	218.7 (18.4)	219.2 (18.9)	217.0 (19.9)	0.33
Arteriole-to-venule ratio	0.70 (0.06)	0.69 (0.06)	0.69 (0.06)	0.080

Abbreviations: dp-ucMGP, desphospho-uncarboxylated matrix Gla protein. Values are means (SD). Baseline refers to the date of blood collection for dp-ucMGP measurement. To convert dp-ucMGP from μg/L into pmol/L, multiply by 94.299. *P-*values denote the significance of the difference in means (ANOVA) across tertiles of the distribution of dp-ucMGP.

### Association with retinal microcirculation

**Figure 2:**
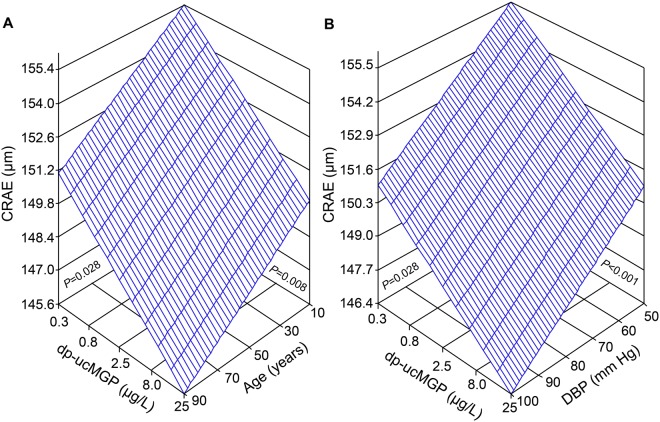
Multivariable-adjusted associations of central retinal arteriolar equivalent (CRAE) with circulating desphospho-uncarboxylated matrix Gla protein (dp-ucMGP) and age (**A**) and diastolic blood pressure (**B**). The plotted planes were standardised to the midpoints of the distributions (means or ratios) of sex, baseline covariables including body mass index, serum total and HDL cholesterol, diabetes mellitus, smoking and drinking, antihypertensive drug treatment (by drug class) and history of cardiovascular disease, follow-up duration and central retinal venular equivalent. The association in panel A was additionally standardised for diastolic blood pressure and in panel B for age.

While accounting for clustering within families (Table 3), a doubling of dp-ucMGP was associated with a 1.40 µm (95% confidence interval [CI], 0.32 to 2.48; *P* = 0.011) smaller CRAE. With adjustments applied for sex and age, the association size was −0.94 µm (CI, −1.84 to −0.05; *P* = 0.040). In a model additionally adjusted for baseline body mass index, diastolic blood pressure, serum total cholesterol and HDL cholesterol, diabetes mellitus, smoking and drinking and use of antihypertensive drugs (by class), history of cardiovascular disease and duration of follow-up and CRVE (Fig. 2 and Table 3), this estimate was −1.03 µm (CI, −1.96 to −0.11; *P* = 0.028). In sensitivity analyses, we additionally accounted for changes from baseline to follow-up in body mass index, diastolic blood pressure, serum total and HDL cholesterol, and we replaced baseline antihypertensive drug treatment by three indicator variables coding for starting, stopping, or continuing blood pressure lowering treatment. A doubling of dp-ucMGP was then associated with a 0.91 µm smaller CRAE (CI, 0.01 to 1.82; *P* = 0.048).

**Table 3:** Association of retinal microvascular parameters and matrix Gla protein.

Model	Central Retinal Arteriolar Calibre		Central Retinal Venular Calibre		Arteriole-to-venule Ratio	
	Estimate (95%CI)	*P*	Estimate (95%CI)	*P*	Estimate (95%CI)	*P*
Unadjusted	−1.40 (−2.48 to −0.32)	0.011	−0.13 (−1.62 to 1.36)	0.86	−0.006 (−0.011 to −0.001)	0.016
Model 1	−0.94 (−1.84 to −0.05)	0.040	1.06 (−0.21 to 2.32)	0.10	−0.005 (−0.010 to −0.0002)	0.043
Model 2	−1.03 (−1.96 to −0.11)	0.028	1.83 (0.55 to 3.11)	0.005	−0.007 (−0.011 to −0.002)	0.007
Model 3	−0.91 (−1.82 to −0.01)	0.048	1.68 (0.40 to 2.96)	0.010	−0.006 (−0.011 to −0.001)	0.014

Association sizes (95% confidence interval) express the changes in the retinal indexes associated with a doubling higher matrix Gla protein. All estimates accounted for clustering within families. Model 1 accounted for sex and baseline age. Model 2 were adjusted for sex, the baseline characteristics age, body mass index, diastolic blood pressure, serum total cholesterol and high-density lipoprotein cholesterol, diabetes mellitus, smoking, alcohol consumption and use of antihypertensive drugs by class, history of cardiovascular disease and duration of follow-up. Model 3 additionally accounted for the changes of body mass index, diastolic blood pressure, serum total cholesterol and high-density lipoprotein cholesterol and 3 indicator variables coding for starting, stopping, or continuing antihypertensive drug treatment from baseline to follow-up. For central retinal arteriolar (venular) calibre, we additionally adjusted for central retinal venular (arteriolar) equivalent.

While only accounting for clustering within families (Table 3), the association size between CRVE and dp-ucMGP was −0.13 µm (CI, −1.62 to 1.36; *P* = 0.86). With adjustments applied for sex and age, in a fully adjusted model and in the sensitivity analysis, the association sizes were 1.06 µm (CI, −0.21 to 2.32; *P* = 0.10), 1.83 µm (CI, 0.55 to 3.11; *P* = 0.005), and 1.68 µm (CI, 0.40 to 2.96; *P* = 0.010).

Associations with AVR mirrored those of CRAE (Table 3). A doubling of dp–ucMGP was associated with lower AVR (−0.006; CI, −0.010 to −0.001; *P* = 0.016) in models only accounting for clustering within families (Table 3). With adjustment for sex and age, in a fully adjusted model and in the sensitivity analysis, the association sizes were −0.005 (CI, −0.010 to −0.0002; *P* = 0.043), −0.007 (CI, −0.011 to −0.002; *P* = 0.007), and −0.006 (CI, −0.011 to −0.001; *P* = 0.014), respectively.

## Discussion

To our knowledge, our study is the first longitudinal population survey assessing the association of retinal microvascular traits with circulating levels of dp-ucMGP measured approximately one decade before retinal photography. Our key finding was that even with multiple adjustments applied CRAE and AVR at follow-up were smaller with higher circulating levels of dp-ucMGP at baseline. Our findings are compatible with studies showing expression of MGP in capillaries and small arterioles^15^ and in particular in the glaucoma-relevant tissues of the eye and the retinal microvasculature^16–20^. Our findings underscore the pivotal role of activated MGP in maintaining ocular homeostasis and are in line with our previous report, which showed that high dp-ucMGP predicted adverse health outcomes, including total, non-cancer and cardiovascular mortality in 2318 FLEMENGHO participants followed up for a median of 14.1 years^11^.

A novel *MGP* transgenic mouse model allows visualising angiogenesis- and glaucoma-relevant tissues by natural fluorescence^20^. These mice have normal intra-ocular pressure. The entire retinal vasculature was red fluorescent (vascular smooth muscle cells) and the fluorescence extended into the capillary bed^20^. The retinal and myocardial microvasculature shared an anatomical and physiological homology^21^. Previously, we demonstrated that in normal and diseased human hearts carboxylated and phosphorylated MGP localises in the media and intima of muscular left ventricular microvessels^15^. Paulus and colleagues proposed a novel paradigm implicating the microcirculation in the pathogenesis of diastolic left ventricular dysfunction^22^. We confirmed the role of activated MGP in maintaining myocardial integrity and diastolic left ventricular performance^15^. Our current observations might therefore reflect a generalised vascular condition not only affecting the microcirculation in the eye, the kidney^13,14^ and the heart^15^, but the central elastic arteries as well^12,23,24^.

The TM located in the anterior chamber of the eye is a tiny porous structure composed of connective tissue beams and trabeculae covered by TM cells. The intratrabecular spaces of the TM allow passage of aqueous humour to Schlemm’s canal, a circular endothelium-lined lymphatic-like vessel, located at the basis of the iris, which is connected to the episcleral venous drainage system through an array of around 35 collector channels^25^. The extracellular matrix of the TM contains numerous structural and organisational proteins, including collagens, laminins, elastin, fibronectin, fibrillins and matricellular proteins. MGP, which is abundantly expressed in the TM, takes part in preserving its structural integrity^17,26^. The TM represents the primary site of outflow resistance to aqueous humour. Adjustment of resistance in the TM is the main mechanism by which intra-ocular pressure is regulated^27^. Elevated intra-ocular pressure is the most important risk factor in the development and progression of glaucoma^27^. In mice, MGP is also abundantly expressed in glaucoma-relevant tissues^17,20^, including TM and sclera as well as in the retinal microvasculature^20^, where MGP exhibits anti-calcification and anti-stiffness properties.

In case-control studies patients with glaucoma compared with controls had a lower distensibility of the common carotid artery^23^ or higher aortic pulse wave velocity^24^. Furthermore, previous studies demonstrated association of glaucoma with retinal microvascular traits^28–30^, including generalised arteriolar narrowing^28,29^. Among 3314 participants enrolled in the Blue Mountains Eye Study^28^, 59 (1.8%) had glaucomatous optic nerve damage and 163 (4.9%) had ocular hypertension. Eyes with glaucomatous damage had significantly narrower (183 *vs*. 194 µm; *P* = 0.0001) CRAE than eyes without glaucoma^28^. Among 3019 Asians enrolled in the Singapore Malay Eye Study^29^, CRAE and CRVE were significantly narrower in 127 (4.2%) participants with glaucoma than in those without glaucoma (136.4 µm *vs*. 139.7 µm, *P* < 0.001 and 209.2 *vs*. 219.7 µm, *P* < 0.001, respectively).

Of potential relevance to our current study, is the strong well-documented protein-protein interaction between MGP and bone morphogenetic protein (BMP), including BMP2^31^ and BMP4^32^, whereby bound MGP reduces BMP signalling. BMPs belong to the transforming growth factor β (TGF-β) superfamily. Following signalling activation via BMP type-1 or type-2 receptors, phosphorylated receptor-regulated SMADs form heterodimeric complexes with the common mediator SMAD4 and translocate to the nucleus to regulate gene expression. In the heart, BMP pathways play a pivotal role in the embryogenesis of the left ventricular chamber^33^, the differentiation of cardiac progenitor cells into functional cardiomyocytes^34^, maintenance of the balance between left ventricular growth and apoptosis^35^, initiation of fibrosis^35^, and Ca^2+^ channel remodelling^36^. In analogy with the cardiac findings, previous studies demonstrated that BMP and TGF-β2 signalling pathways are also active in the eye^26,37–39^. Expression of BMP-4 and its receptors plays a pivotal role in the early embryogenesis of the eye^37^. Rat studies also demonstrated expression of BMP4 and its receptors in the adult eye, including the corneal microvascular endothelium^38^. TGF-β2 is associated with increased extracellular matrix deposition (fibronectin) in the TM, which leads to an increased resistance of aqueous humour outflow^39^. In this context^39^, the BMP and TGF-β2 signalling pathways antagonise each other’s antifibrotic and profibrotic actions. Less activated MGP might also be involved, via increased BMP signalling, in higher arteriolar stiffness and smaller CRAE in the eye.

Several of our observations are in line with the literature and support the validity of our findings^40^. For instance, in the baseline data from the Inter99 Eye Study, CRAE was 0.2 µm smaller for each unit increase in age or systolic blood pressure and 4.0 µm larger in smokers compared with non-smokers^40^. The corresponding estimates in our current study were: −0.2 µm, −0.2 µm and +3.5 µm, respectively. On the other hand, our current study must also be interpreted within the context of its potential limitations. First, we did not take retinal photographs at baseline, so that we could not assess whether MGP is a modulator of retinal arteriolar narrowing in relation to age, blood pressure, body mass index or other risk factors. Second, an observational study cannot assess causality. However, our study satisfies the Bradford-Hill criteria of temporality (a baseline marker predicting a trait of interest), plausibility and coherence (between clinical and experimental observations). Nevertheless, further experimental and clinical studies are required to substantiate our current observations. MGP staining studies using conformation-specific MGP antibodies^15^ to identify the exact localisation of MGP in the human TM and the retinal microvasculature is one possible approach. Third, in view of the prevalence of glaucoma in European populations estimated to run at a rate of approximately 3%^41^ and our limited sample size, we could not ascertain the association of symptomatic glaucoma with arteriolar retinal narrowing or circulating dp-ucMGP. Fourth, we did not measure circulating levels of vitamin K, which is rarely done in research or clinical practice, because of the complexity of the assay and the lack of a high-throughput method^42^ and because plasma levels only reflect dietary intake (vitamin K_1_; phylloquinone) and production by the gut microflora (vitamin K_2_; menaquinones) without giving any indication of functionality, i.e. the amount of MGP undergoing carboxylation^9^. Finally, our current findings in white Flemish cannot be extrapolated to other ethnicities.

Notwithstanding potential limitations, our findings may have important clinical implications. High levels of plasma dp-ucMGP are a proxy for vitamin K deficiency^9,10^. Levels ranging from 1.4 to 4.6 μg/L are probably optimal in terms of the risk of mortality and macrovascular cardiovascular complications^11^. In Flemish, the 4.6 μg/L threshold corresponds with the 65th percentile of the dp-ucMGP distribution, indicating that nearly 35% of Flemish might be vitamin K deficient. Vitamin K supplementation reduced aortic pulse wave velocity in healthy postmenopausal women^10^. Assuming reversibility, our current findings extend the protective role of vitamin K to the retinal microcirculation and TM. Vitamin K has a very wide safety range. Sources are leafy vegetables (phylloquinone; vitamin K_1_), fermented foods (menaquinones; vitamin K_2_) or dietary supplements.

In the general population, CRAE and AVR at follow-up were smaller with higher levels of circulating inactive dp-ucMGP at baseline, a biomarker of vitamin K deficiency. Our study highlight the possibility that vitamin K supplementation might promote ocular health. Further studies should clarify the underlying molecular pathways and substantiate the speculation that vitamin K supplementation might promote ocular health and prevent glaucoma-induced optic nerve damage.

## Methods

### Study population

The Ethics Committee of the University of Leuven approved the FLEMENGHO protocol^11^. FLEMENGHO complies with the Helsinki declaration for research in humans^43^. At each contact, participants gave or renewed informed written consent. FLEMENGHO is a family-based population study, for which recruitment started in 1985^11,14^. Of 3343 participants, 1285 underwent retinal photography (2008–2015). The participation rate was 78.0% at enrolment and 76.0% for retinal photography. In the context of this article, baseline refers to blood sampling for the measurement of dp-ucMGP (1996–2010) and follow-up to retinal imaging (2008–2015). We excluded participants from analysis if the retinal photographs were of too low quality to be reliably graded (n = 221) or if baseline plasma dp-ucMGP (n = 106) or biochemical (n = 7) measurements were missing. This left 951 participants with plasma dp-ucMGP measured at baseline, with gradable retinal photographs at follow-up, and with all covariables available at baseline and follow-up. Finally, we excluded participants from analysis, if they were taking warfarin (n = 1), or if retinal microvascular diameters (n = 8), plasma dp-ucMGP (n = 3) or blood pressure (n = 4) were more than 3 SDs away from the population mean. Thus, the number of participants statistically analysed totalled 935.

### Retinal photography

Participants were asked to refrain from heavy exercise, smoking, drinking alcohol or caffeine-containing beverages for at least 3 hours prior to retinal imaging. We applied a non-mydriatic approach in a dimly lit room to acquire retinal photographs, one image per eye in each participant, with the Canon Cr-DGi retinal visualisation system combined with the Canon D 50 digital camera (Canon Inc, Medical Equipment Group, Utsunomiya, Japan). We measured the CRAE and CRVE equivalent, which represent the retinal arteriolar and venular diameter. We used the validated computer-assisted programme IVAN (Vasculomatic ala Nicola, version 1.1, Department of Ophthalmology and Visual Science, University of Wisconsin-Madison, Madison, WI) based on formulae published by Parr^44^ and Hubbard^45^. The IVAN software returns average vessel diameters according to the revised Knudtson formula^46^. The AVR was CRAE divided by CRVE. For analysis, we averaged each participant’s measurements at both eyes. Intra-observer variability according to the Bland and Altman method^47^ was 11.7% for CRAE, 9.6% for CRVE and 12.5% for AVR1. The corresponding estimates for interobserver variability were 10.8%, 9.9% and 14.6%, respectively^1^.

### Baseline clinical and biochemical measurements

Blood pressure was the average of five consecutive auscultatory readings obtained with a standard mercury sphygmomanometer. Hypertension was a blood pressure of at least 140 mm Hg systolic or 90 mm Hg diastolic or use of antihypertensive drugs. The study nurses also administered questionnaires inquiring into each participant’s medical history, smoking and drinking habits, and intake of medications. At baseline and follow-up, fasting blood samples were analysed for plasma glucose, serum total and HDL cholesterol and serum creatinine, using automated methods in a single certified laboratory. dp-ucMGP was measured on citrated plasma by pre-commercial ELISA kits at VitaK (Maastricht University, The Netherlands)^48^. This dual-antibody MGP assay performed satisfactory with respect to intra-assay (5.6%) and inter-assay (9.9%) variation and the detection limit (0.22 μg/L)^48^. Diabetes mellitus was a fasting plasma glucose of 7.0 mmol/L (126 mg/dL) or higher or use of antidiabetic agents.

### Statistical analyses

For database management and statistical analysis, we used SAS software, version 9.4 (SAS Institute Inc., Cary, NC). We compared means and proportions by the large-sample z-test or ANOVA and by the χ^2^-statistic, respectively. We normalised the distributions of dp-ucMGP by a logarithmic transformation. Statistical significance was a two-sided significance of 0.05.

In unadjusted and multivariable-adjusted analyses, we expressed association sizes between the retinal phenotypes at follow-up and baseline dp-ucMGP for a doubling of the biomarker. As in previous publications^49,50^, we adjusted for sex and the baseline covariables age, body mass index, diastolic blood pressure, serum total and HDL cholesterol, diabetes mellitus, smoking and drinking and antihypertensive drug treatment, broken down into diuretics (thiazides, loop diuretics and aldosterone antagonists), inhibitors of the renin-angiotensin system (β-blockers, angiotensin-converting enzyme inhibitors and angiotensin type-1 receptor blockers), vasodilators (calcium-channel blockers and α-blockers), history of cardiovascular disease and follow-up duration. In sensitivity analyses, we additionally accounted for changes in body mass index, diastolic blood pressure, serum total and HDL cholesterol, and three indicator variables coding for starting, stopping, or continuing antihypertensive drug treatment from baseline to follow-up. For CRAE, we additionally adjusted for CRVE. The final multivariable-adjusted analyses relied on mixed models as implemented in SAS 9.4, which accounted for family clusters modelled as a random effect and the other covariables modelled as fixed effects.

## Data Availability

The corresponding author will make anonymized data available to researchers who present an outstanding research plan that will move the field forward.
